# Efficacy and safety of immune checkpoint inhibitor plus bevacizumab-based chemotherapy in advanced or recurrent *KRAS*-mutant non-squamous NSCLC: the SPIRAL-STONE study

**DOI:** 10.3389/fimmu.2026.1875501

**Published:** 2026-10-12

**Authors:** Masaki Ishida, Tadaaki Yamada, Riko Kamada, Makoto Hibino, Masashi Egami, Takayuki Nakano, Tatsuya Imabayashi, Chieko Takumi, Koichi Ishida, Masafumi Takeshita, Ken Masubuchi, Miiru Izumi, Takayo Ota, Shoki Morito, Yusuke Chihara, Soichi Hirai, Shinsuke Shiotsu, Ryo Sawada, Hayato Kawachi, Naoya Nishioka, Masahiro Iwasaku, Koichi Takayama

**Affiliations:** 1 Department of Pulmonary Medicine, Graduate School of Medical Science, Kyoto Prefectural University of Medicine, Kyoto, Japan; 2 Department of Respiratory Medicine, Shonan Fujisawa Tokushukai Hospital, Kanagawa, Fujisawa, Japan; 3 Department of Respiratory Medicine, Shonan Oiso Hospital, Kanagawa, Oiso, Japan; 4 Department of Respiratory Medicine, Fukuchiyama City Hospital, Kyoto, Japan; 5 Department of Respiratory Medicine, Japanese Red Cross Kyoto Daiichi Hospital, Kyoto, Japan; 6 Department of Respiratory Medicine, Ichinomiya Nishi Hospital, Aichi, Ichinomiya, Japan; 7 Division of Respiratory Medicine, Gunma Prefectural Cancer Center, Ota, Gunma, Japan; 8 Department of Respiratory Medicine, National Hospital Organization Omuta National Hospital, Omuta, Fukuoka, Japan; 9 Department of Breast Medical Oncology, Izumi City General Hospital, Izumi, Japan; 10 Department of Respiratory Medicine, Uji-Tokushukai Medical Center, Kyoto, Japan; 11 Department of Respiratory Medicine, Japanese Red Cross Kyoto Daini Hospital, Kyoto, Japan

**Keywords:** ICI plus bevacizumab-based chemotherapy, *KRAS*, non-small cell lung cancer, prospective observational study, VEGF

## Abstract

**Introduction:**

Kirsten rat sarcoma viral oncogene homolog (*KRAS*)-mutant lung cancer is associated with a poor prognosis, underscoring the need for effective pharmacological treatments. An exploratory subgroup analysis of IMpower150 suggested potential clinical benefit of the atezolizumab-containing regimen in KRAS-mutant non-squamous NSCLC, although prospective data remain limited. We prospectively evaluated its efficacy and safety and performed exploratory biomarker analyses.

**Methods:**

The study cohort comprised patients with advanced or recurrent *KRAS*-mutant non-squamous NSCLC scheduled to receive ICI plus bevacizumab-based chemotherapy at 10 Japanese institutions between January 2023 and December 2024. The primary endpoint was progression-free survival (PFS). Secondary endpoints included disease control rate (DCR), objective response rate (ORR), overall survival (OS), and safety. Associations between baseline serum levels of vascular endothelial growth factor (VEGF)-A and VEGF-D and treatment outcomes were explored in patients with available pretreatment samples.

**Results:**

Of 22 enrolled patients, 21 received treatment and were analyzed for efficacy and safety; 17 were analyzed for serum biomarkers. All patients received atezolizumab, bevacizumab, carboplatin, and paclitaxel. The median PFS and OS were 6.0 and 17.1 months, respectively. Among 19 patients with measurable disease, the ORR was 42.1%, and the DCR was 68.4%. Grade ≥3 hematologic and non-hematologic adverse events occurred in 33.3% and 47.6% of patients, respectively. Baseline serum VEGF-A and VEGF-D levels were not significantly associated with the treatment outcomes.

**Conclusions:**

ICI plus bevacizumab-based chemotherapy showed an ORR of 42.1%, a median PFS of 6.0 months, and a median OS of 17.1 months in patients with *KRAS*-mutant non-squamous NSCLC. Given the small sample size and single-arm design, these findings should be considered descriptive and hypothesis-generating. Pretreatment serum VEGF-A and VEGF-D levels were not significantly associated with clinical outcomes, although limited statistical power precludes definitive conclusions. Larger comparative studies with comprehensive molecular profiling are warranted.

## 1 Introduction

Pharmacotherapy for advanced non-small cell lung cancer (NSCLC) has substantially evolved with the advent of personalized medicine, including the discovery of oncogenic driver alterations, targeted therapies, and immune checkpoint inhibitors (ICIs), thereby improving patient prognosis (1). Among these, Kirsten rat sarcoma viral oncogene homolog (*KRAS*) mutations are among the most common oncogenic driver alterations in NSCLC, occurring in approximately 25%–30% and 10%–15% of patients in Western and East Asian populations, respectively (2–4).

*KRAS*-mutant NSCLC is increasingly recognized as a biologically and clinically heterogeneous disease rather than a single molecular entity (5–8). Its clinical characteristics and therapeutic sensitivity vary according to the specific *KRAS* mutation subtype and co-occurring genomic alterations, particularly those involving *STK11*, *KEAP1*, and *TP53*. These molecular features are associated with distinct tumor immune microenvironments and may influence prognosis and responses to ICIs. The development of *KRAS* G12C inhibitors has expanded treatment options for patients with previously treated *KRAS* G12C-mutant NSCLC (9, 10). However, *KRAS* G12C inhibitors are not currently established as standard first-line therapy, and effective targeted therapies remain limited for most non-G12C variants.

In pivotal trials of patients with advanced non-squamous NSCLC without epidermal growth factor receptor (*EGFR*) or anaplastic lymphoma kinase (*ALK*) alterations, the addition of an ICI to bevacizumab-based chemotherapy improved clinical outcomes compared with bevacizumab plus chemotherapy alone (11, 12). However, the contemporary first-line treatment landscape for *KRAS*-mutant NSCLC includes several ICI-based strategies, and treatment selection is influenced by PD-L1 expression, patient characteristics, disease burden, *KRAS* mutation subtype, and co-occurring genomic alterations (5, 7, 8). An exploratory subgroup analysis of IMpower150 suggested that the atezolizumab-containing regimen may provide clinical benefit in patients with *KRAS*-mutant NSCLC (5). Nevertheless, these findings were derived from subgroup or indirect analyses and do not establish the superiority of this regimen over other first-line treatment options. Prospective data specifically evaluating the efficacy and safety of ICI plus bevacizumab-based chemotherapy in a molecularly defined *KRAS*-mutant population remain limited.

Vascular endothelial growth factor (VEGF) signaling regulates both tumor angiogenesis and the immunosuppressive tumor microenvironment (13). Identifying biomarkers that predict response to anti-VEGF therapy combined with chemoimmunotherapy in NSCLC remains an important clinical challenge. Although circulating angiogenic factors, such as VEGF-A and VEGF-D, have been investigated as potential biomarkers for antiangiogenic therapy (14, 15), their clinical relevance may vary depending on the antiangiogenic agent, treatment line, combination regimen, and patient population. In particular, it remains unclear whether pretreatment serum VEGF-A or VEGF-D levels are associated with clinical outcomes in patients with *KRAS*-mutant NSCLC treated with ICI plus bevacizumab-based chemotherapy.

In this prospective multicenter study, we evaluated the efficacy and safety of ICI plus bevacizumab-based chemotherapy in patients with advanced or recurrent *KRAS*-mutant non-squamous NSCLC. We also performed an exploratory analysis to assess the correlation between pretreatment serum VEGF-A and VEGF-D levels and treatment outcomes in this molecularly defined population.

## 2 Material and methods

### 2.1 Study design and patients

Patients diagnosed with advanced or recurrent *KRAS*-mutant non-squamous NSCLC who were scheduled to receive ICI plus bevacizumab-based chemotherapy at 10 institutions in Japan were prospectively enrolled between January 2023 and December 2024. All patients received atezolizumab plus bevacizumab, carboplatin, and paclitaxel (ABCP) as first-line systemic therapy for advanced or recurrent disease. None of the patients had received prior systemic anticancer therapy, including perioperative systemic therapy, or radiotherapy before initiation of the study treatment. The planned sample size was 30 patients, based on enrollment feasibility in routine clinical practice. No formal statistical power calculation was performed because this was an exploratory prospective observational study. The target sample size was therefore determined pragmatically rather than on the basis of a prespecified treatment effect or biomarker hypothesis. However, enrollment was closed at 22 patients because no additional eligible patients could be recruited during the predefined enrollment period. Patients were eligible if they met all of the following criteria: (1) a diagnosis of unresectable stage III, stage IV, or postoperative recurrent non-squamous NSCLC according to the American Joint Committee on Cancer Staging Manual, version 8, (2) age ≥20 years at the time of informed consent, (3) presence of a *KRAS* mutation, (4) planned treatment with an ICI plus bevacizumab-based chemotherapy, and (5) provision of written informed consent prior to study registration. Those with unresectable stage III disease were not candidates for definitive chemoradiotherapy. Patients harboring *EGFR* mutations or *ALK* rearrangements, as well as those deemed unsuitable for study participation by the treating physician after careful clinical review, were excluded. *KRAS* mutation status was determined using tumor tissue specimens by centralized molecular testing. An Oncomine-based assay was used in 15 patients, whereas an AmoyDx-based assay was used in the remaining six patients. Oncomine testing enabled identification of individual *KRAS* variants, whereas the AmoyDx assay reported certain variants, including G12A, G12V, G12R, and G13C, collectively as “*KRAS* Others”. Therefore, the individual subtype could not be further specified for patients reported in this category. Other actionable driver alterations included in the respective assay panels were also evaluated, and no actionable alterations other than *KRAS* mutations were identified. *STK11*, *KEAP1*, and *TP53* alterations were not assessed in this study. The data cutoff date was December 31, 2025. The median follow-up time was estimated using the reverse Kaplan–Meier method. Each patient’s medical record was prospectively reviewed. The following data were collected: age, sex, Eastern Cooperative Oncology Group performance status (ECOG-PS), smoking status, tumor histology, disease stage, metastatic sites (bone, malignant pleural effusion [MPE], liver, and brain), *KRAS* mutation subtype, programmed death-ligand 1 (PD-L1) tumor proportion score (TPS), treatment regimens (induction and maintenance), treatment discontinuation and its reasons, subsequent systemic therapies, adverse events (AEs), and follow-up data, including treatment initiation, disease progression, death, and final follow-up. PD-L1 expression was assessed at SRL, Inc. using the PD-L1 IHC 22C3 pharmDx assay (Agilent Technologies, Santa Clara, CA, USA), and TPS was recorded.

AEs were graded according to the Common Terminology Criteria for Adverse Events version 5.0. The relationship between each AE and ABCP was assessed by the treating investigator.

Clinical outcomes included the objective response rate (ORR), disease control rate (DCR), maximum percent change from baseline in the sum of target lesion diameters, progression-free survival (PFS), and overall survival (OS). Baseline laboratory values were collected to calculate the neutrophil-to-lymphocyte ratio (NLR), Glasgow Prognostic Score (GPS), and prognostic nutritional index (PNI). Baseline serum VEGF-A and VEGF-D levels were measured in patients with available pretreatment serum samples. The efficacy population comprised all patients who received at least one dose of ABCP, and PFS and OS were evaluated in this population. The safety population likewise comprised all patients who received at least one dose of ABCP. The response-evaluable population comprised patients in the efficacy population with measurable disease at baseline, in whom ORR and DCR were assessed. The biomarker population comprised patients in the efficacy population with available pretreatment serum samples. The primary endpoint was PFS in the efficacy population. Secondary endpoints included OS, ORR, DCR, and safety outcomes in the corresponding analysis populations described above.

This study was conducted in accordance with the Declaration of Helsinki of the World Medical Association (16). The study protocol was approved by the Institutional Review Board of Kyoto Prefectural University of Medicine (ERB-C-2692-6) and each participating hospital and was registered at the University Hospital Medical Information Network (UMIN000049780; study information disclosed on January 4, 2023), before enrollment of the first patient. The manuscript was prepared in accordance with the STROBE reporting guideline for observational studies. All patients provided written informed consent before participation.

### 2.2 Treatment

All patients received atezolizumab 1,200 mg/body, bevacizumab 15 mg/kg, carboplatin at an area under the curve of 6, and paclitaxel 200 mg/m² intravenously on day 1 of each 3-week cycle. Because this was an observational study, the number of induction cycles was not mandated by the study protocol and was determined by the treating physician in routine clinical practice. Four to six induction cycles could be administered according to the prescribing information and the patient’s clinical condition. Patients without disease progression or unacceptable toxicity could subsequently receive maintenance atezolizumab plus bevacizumab every 3 weeks. Treatment modification, interruption, or discontinuation was determined by the treating physician according to the prescribing information for each agent and the patient’s clinical condition.

### 2.3 Efficacy analysis

PFS was defined as the time from initiation of ICI plus bevacizumab-based chemotherapy to the first documentation of radiologic or clinical disease progression or death from any cause, whichever occurred first. Patients without documented progression were censored at the last date on which they were confirmed to be progression-free. Treatment discontinuation or initiation of subsequent systemic therapy without documented progression did not constitute a PFS event or censoring event. OS was defined as the time from initiation of ICI plus bevacizumab-based chemotherapy to death from any cause.

Tumor response was assessed by investigators at each institution in patients with measurable disease at baseline according to the Response Evaluation Criteria in Solid Tumors version 1.1. Because this was an observational study, the timing of radiologic assessments was not protocol-mandated; computed tomography or magnetic resonance imaging every 2–3 months was recommended during the observation period. The dates of individual response assessments were not prospectively captured in sufficient detail to reconstruct the actual imaging intervals reliably. Confirmatory imaging for complete or partial response was not required, and no minimum duration was prespecified for stable disease. The ORR was defined as the proportion of patients who achieved a complete or partial response as the best overall response. DCR was defined as the proportion of patients who achieved complete response, partial response, or stable disease.

The maximum percentage change from baseline in the sum of the target lesion diameters was calculated among patients with measurable target lesions at baseline.

### 2.4 Assessments of serum VEGF-A and VEGF-D

Baseline serum samples were prospectively collected before treatment initiation. Blood collection and serum processing were standardized across participating institutions. After collection, blood was allowed to clot at room temperature for 30 minutes and centrifuged at 1,000 × g for 15 minutes. The separated serum was aliquoted, frozen, and stored at −80 °C until analysis. The analyses of serum VEGF-A and VEGF-D were not prespecified in the study protocol and were conducted as exploratory biomarker analyses. VEGF-D was evaluated based on preclinical evidence suggesting that it may contribute to alternative angiogenic signaling when VEGF-A-mediated angiogenesis is inhibited (17, 18). Because bevacizumab specifically neutralizes VEGF-A but does not directly inhibit VEGF-D, circulating VEGF-D was examined as an exploratory marker of VEGF-pathway activity outside the direct target spectrum of bevacizumab.

Serum VEGF-A and VEGF-D concentrations were measured using commercially available enzyme-linked immunosorbent assay (ELISA) kits. VEGF-A was measured using the Human VEGF-A ELISA Kit (RayBiotech, Peachtree Corners, GA, USA; catalog number ELH-VEGF), and VEGF-D was measured using the Human VEGF-D ELISA Kit (Aviva Systems Biology, San Diego, CA, USA; catalog number OKRC01017). Samples were measured in duplicate after two-fold dilution, and the mean of the duplicate measurements was used for analysis. All samples were analyzed in a single assay batch and underwent only one freeze–thaw cycle before measurement. All assays were performed according to the manufacturers’ instructions. According to the manufacturers’ specifications, the intra-assay and inter-assay coefficients of variation were <10% and <12%, respectively. All measured concentrations fell within the ranges of the standard curves used for quantification, and no samples required extrapolation beyond the standard curves.

### 2.5 Statistical analyses

Statistical tests were two-sided, and statistical significance was set at p<0.05. Continuous variables are summarized as medians (ranges) and were compared using the Wilcoxon rank-sum test. Categorical variables were compared using the chi-square or Fisher exact test, as appropriate. The ORR and DCR were reported with 95% confidence intervals (CIs) estimated using the Wilson method.

Baseline serum VEGF-A and VEGF-D levels were dichotomized using the sample median as the cutoff. The sample medians were 0.35 ng/mL for VEGF-A and 1.28 ng/mL for VEGF-D. Values equal to or above the sample median were classified as above-median, whereas values below the median were classified as below-median. As sensitivity analyses, baseline serum VEGF-A and VEGF-D levels were also evaluated as natural log-transformed continuous variables. Cox proportional hazards models were used to assess their associations with PFS and OS. Hazard ratios were expressed per doubling of the biomarker level and were calculated as exp[β × ln(2)], where β represents the Cox regression coefficient.

Because of the limited sample size and number of outcome events, adjusted analyses included only smoking status as a potential confounder, and separate models were constructed for VEGF-A and VEGF-D to reduce overfitting. The correlation between baseline serum VEGF-A and VEGF-D levels was assessed using Spearman’s rank correlation coefficient. Survival curves were calculated using the Kaplan–Meier method, and differences were compared using the log-rank test.

NLR was calculated as the neutrophil count divided by the lymphocyte count. GPS was calculated using serum C-reactive protein and albumin levels, and PNI was calculated using serum albumin and peripheral lymphocyte counts. In the Cox analyses, NLR, GPS, and PNI were dichotomized using prespecified cutoffs based on previous studies (NLR ≥3, GPS ≥1, and PNI ≥45.5) (19–21). Univariable Cox proportional hazards models estimated hazard ratios (HRs) and 95% CIs for PFS and OS. The proportional-hazards assumption was assessed using Schoenfeld residuals. All biomarker analyses were exploratory, and the corresponding p-values were interpreted descriptively without adjustment for multiple comparisons.

A swimmer plot was used to illustrate the individual treatment courses, including the best overall response and durations of the induction and maintenance phases. Key clinical events were annotated, including disease progression, death, final follow-up, ongoing treatment at the final follow-up, and AE-related treatment discontinuation, with the corresponding AE specified. Statistical analyses were performed using R version 4.5.1 (R Foundation for Statistical Computing, Vienna, Austria) and GraphPad Prism version 10.6.1 (GraphPad Software, San Diego, CA, USA).

## 3 Results

### 3.1 Patient characteristics

Twenty-two patients with advanced or recurrent *KRAS*-mutant, non-squamous NSCLC were enrolled. One patient was excluded because ICI plus bevacizumab-based chemotherapy was not initiated owing to hemoptysis. All patients received atezolizumab plus bevacizumab, carboplatin, and paclitaxel (ABCP). Four additional patients were excluded from the serum analysis because pretreatment blood samples were not collected. Accordingly, 21 patients comprised both the efficacy and safety populations. Among these, 19 patients with measurable disease at baseline comprised the response-evaluable population, and 17 patients with available pretreatment serum samples comprised the biomarker population (**Figure 1**). At the data cutoff date, the median follow-up time was 25.7 months (95% CI, 17.7–29.8 months). Baseline patient characteristics are summarized in **Table 1**. The median age was 73 years (range, 48–81), and 8 patients (38.1%) were aged ≥75 years. Of the 21 patients, 14 (66.7%) were male, 11 (52.4%) had an ECOG-PS of 0, 2 (9.5%) had unresectable stage III disease, 14 (66.7%) had stage IV disease, and 5 (23.8%) had postoperative recurrence. Bone metastasis was observed in 10 patients (47.6%), MPE in 8 (38.1%), liver metastasis in 3 (14.3%), and brain metastasis in 3 (14.3%). *KRAS* mutation subtypes included G12C in 9 patients (42.9%), G12D in 3 (14.3%), G12A in 2 (9.5%), and G12V in 1 (4.8%). PD-L1 TPS ≥50% was observed in six patients (28.6%), and 17 (81.0%) had a history of smoking.

**Figure 1:**
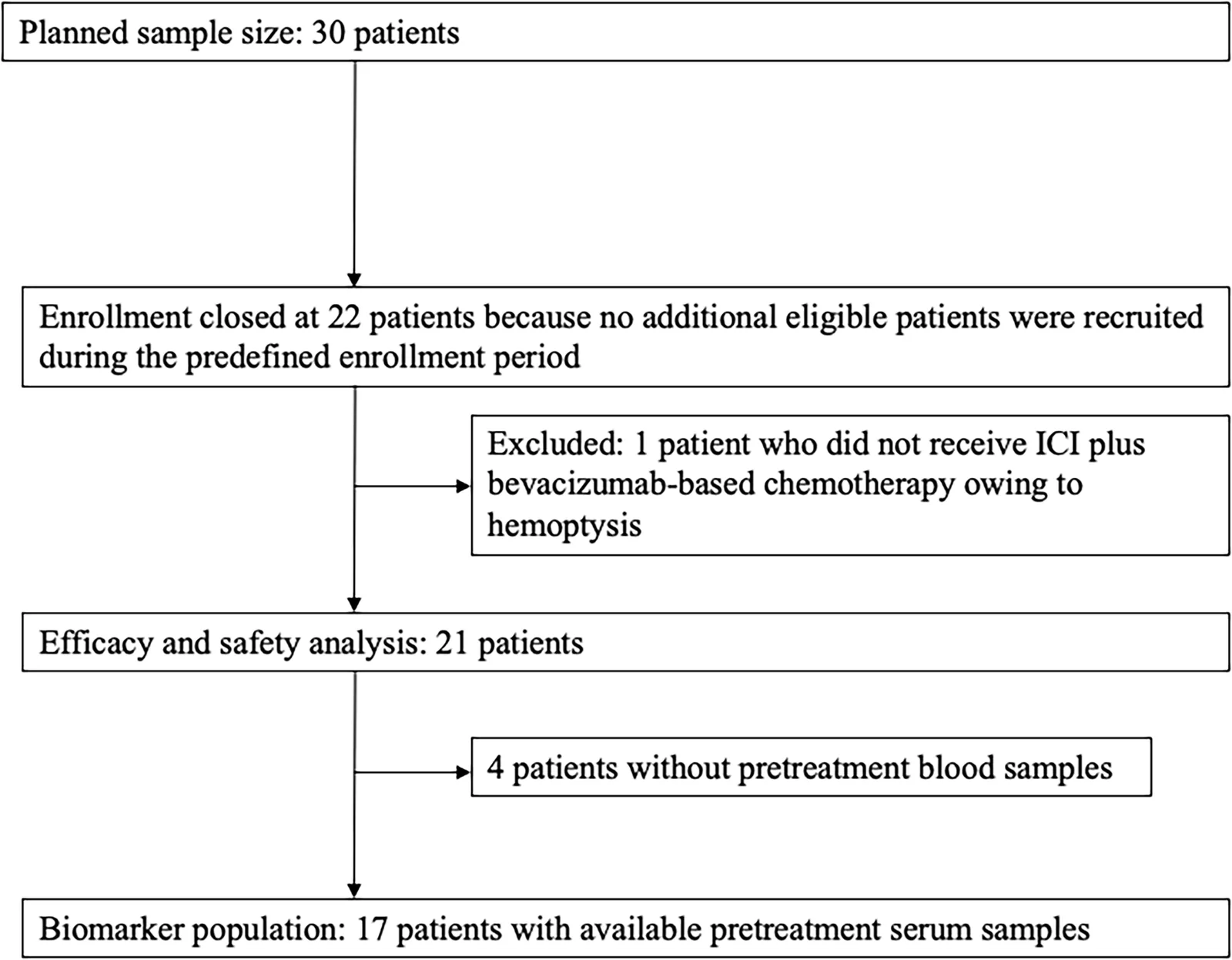
Patient flow diagram of this study. *KRAS*, Kirsten rat sarcoma viral oncogene homolog; NSCLC, non-small cell lung cancer; ICI, immune checkpoint inhibitor.

**Table 1:** Baseline characteristics of the efficacy population (n = 21).

Characteristics	Efficacy population (n = 21)
Age	
Median (range)	73 (48–81)
≥75	8 (38.1%)
Sex	
Male	14 (66.7%)
Female	7 (33.3%)
ECOG-PS	
0	11 (52.4%)
1	8 (38.1%)
2	2 (9.5%)
Tumor histology	
Adenocarcinoma	20 (95.2%)
Pleomorphic carcinoma	1 (4.8%)
Stage	
Unresectable stage III	2 (9.5%)
Stage IV	14 (66.7%)
Postoperative recurrence	5 (23.8%)
Metastasis site	
Bone	10 (47.6%)
Malignant pleural effusion	8 (38.1%)
Liver	3 (14.3%)
Brain	3 (14.3%)
*KRAS* mutation subtype	
G12C	9 (42.9%)
G12D	3 (14.3%)
G12A	2 (9.5%)
G12V	1 (4.8%)
Others	6 (28.6%)
PD-L1 TPS	
≥50%	6 (28.6%)
1–49%	8 (38.1%)
<1%	7 (33.3%)
Smoking status	
Current/Former	17 (81.0%)
Never	4 (19.0%)

Others: variants reported collectively as “*KRAS* Others” by the AmoyDx assay, including G12A, G12V, G12R, and G13C; individual subtypes were not distinguishable using this assay.

ECOG-PS, Eastern Cooperative Oncology Group performance status; *KRAS*, *Kirsten rat sarcoma viral oncogene homolog;* PD-L1, programmed death-ligand 1; TPS, tumor proportion score.

### 3.2 Clinical efficacy of ABCP in patients with *KRAS*-mutant non-squamous NSCLC

In the response-evaluable population (n=19), the ORR and DCR were 42.1% (8/19; 95% CI, 23.1–63.7) and 68.4% (13/19; 95% CI, 46.0–84.6), respectively (**Figure 2**). In the efficacy population (n=21), 17 PFS events and 11 OS events were observed. The median PFS and OS were 6.0 months (95% CI, 3.7–11.6) and 17.1 months (95% CI, 6.7–not reached), respectively (**Figure 3**). Individual treatment details and clinical courses of patients in the efficacy population are shown in the swimmer plot (**Figure 4**). The median number of induction cycles was 3 (range, 1–4). Eight patients (38.1%) proceeded to maintenance therapy with atezolizumab plus bevacizumab, and the median number of maintenance cycles among these patients was 6.5 (range, 1–16). At the data cutoff, eight patients were still receiving systemic anticancer therapy. The systemic therapies administered after ABCP are summarized in **Supplementary Table 1**. Overall, 13 patients (61.9%) received subsequent systemic anticancer therapy after ABCP, whereas 8 patients (38.1%) received no further systemic therapies. ABCP was discontinued owing to disease progression in 10 patients (47.6%), AEs in 5 (23.8%), and death in 3 (14.3%). One additional patient discontinued ABCP after traumatic subarachnoid hemorrhage, which was unrelated to treatment and was not classified as an adverse event. Two patients (9.5%) achieved a partial response after four induction cycles and did not proceed to maintenance therapy; both maintained their responses during subsequent observation. In both patients, the decision not to proceed to maintenance therapy was based on patient preference rather than treatment-related adverse events. Univariable Cox proportional hazards analyses for PFS and OS were performed to assess the association between clinical factors and outcomes. No baseline factors were significantly associated with PFS. MPE (HR, 4.85; 95% CI, 1.38–16.97; p=0.014) and GPS ≥1 (HR, 4.38; 95% CI, 1.26–15.22; p=0.020) were significantly associated with shorter OS, whereas no other baseline factors were significantly associated with OS (**Supplementary Figure 1**). The proportional-hazards assumption may have been violated for GPS in the univariable PFS model (p=0.010); therefore, its hazard ratio should be interpreted with caution. No evidence of violation was found for the other univariable Cox models.

**Figure 2:**
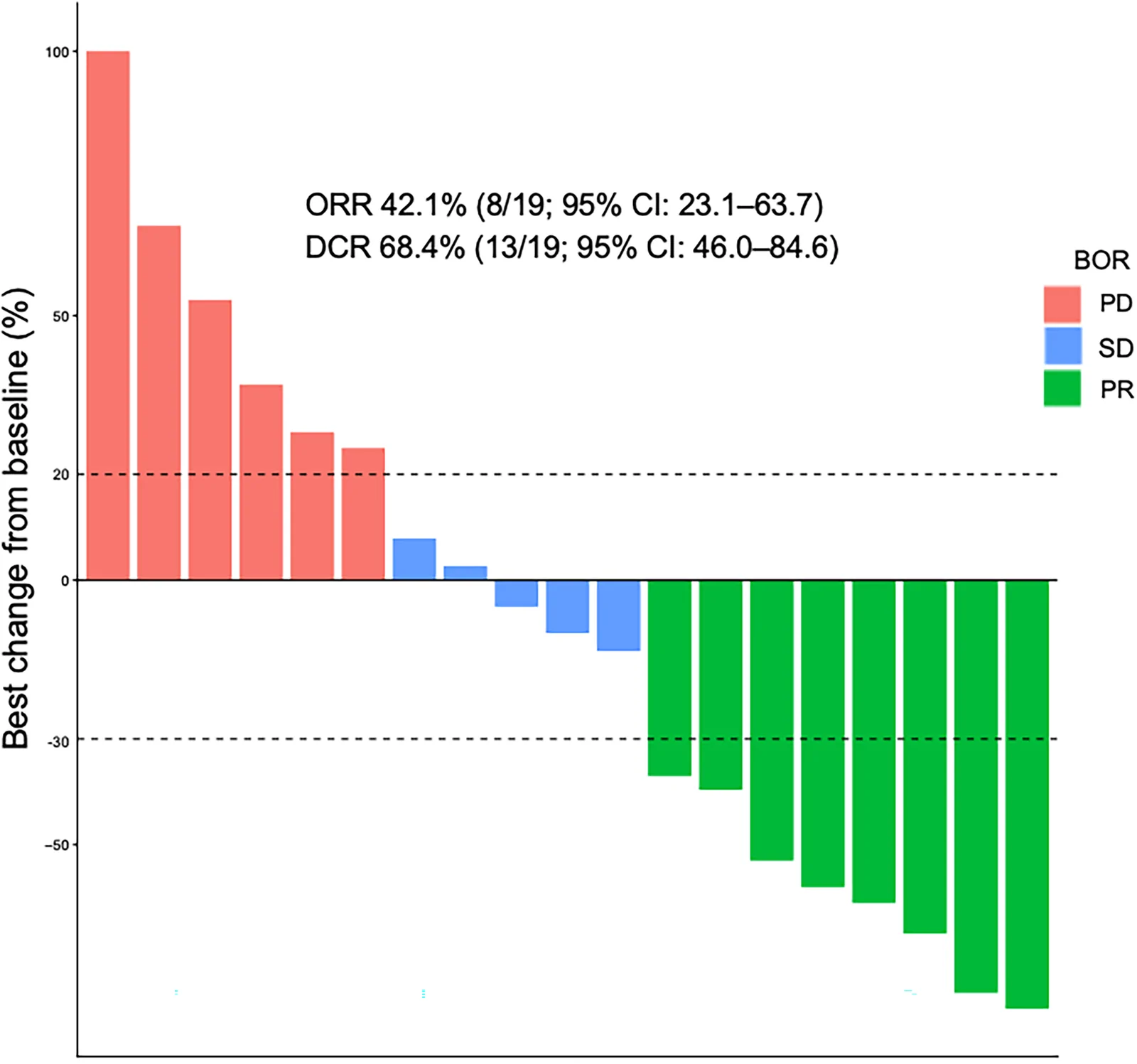
Waterfall plot of the best percentage change from baseline in tumor size. ORR, objective response rate; DCR, disease control rate; CI, confidence interval; BOR, best overall response.

**Figure 3:**
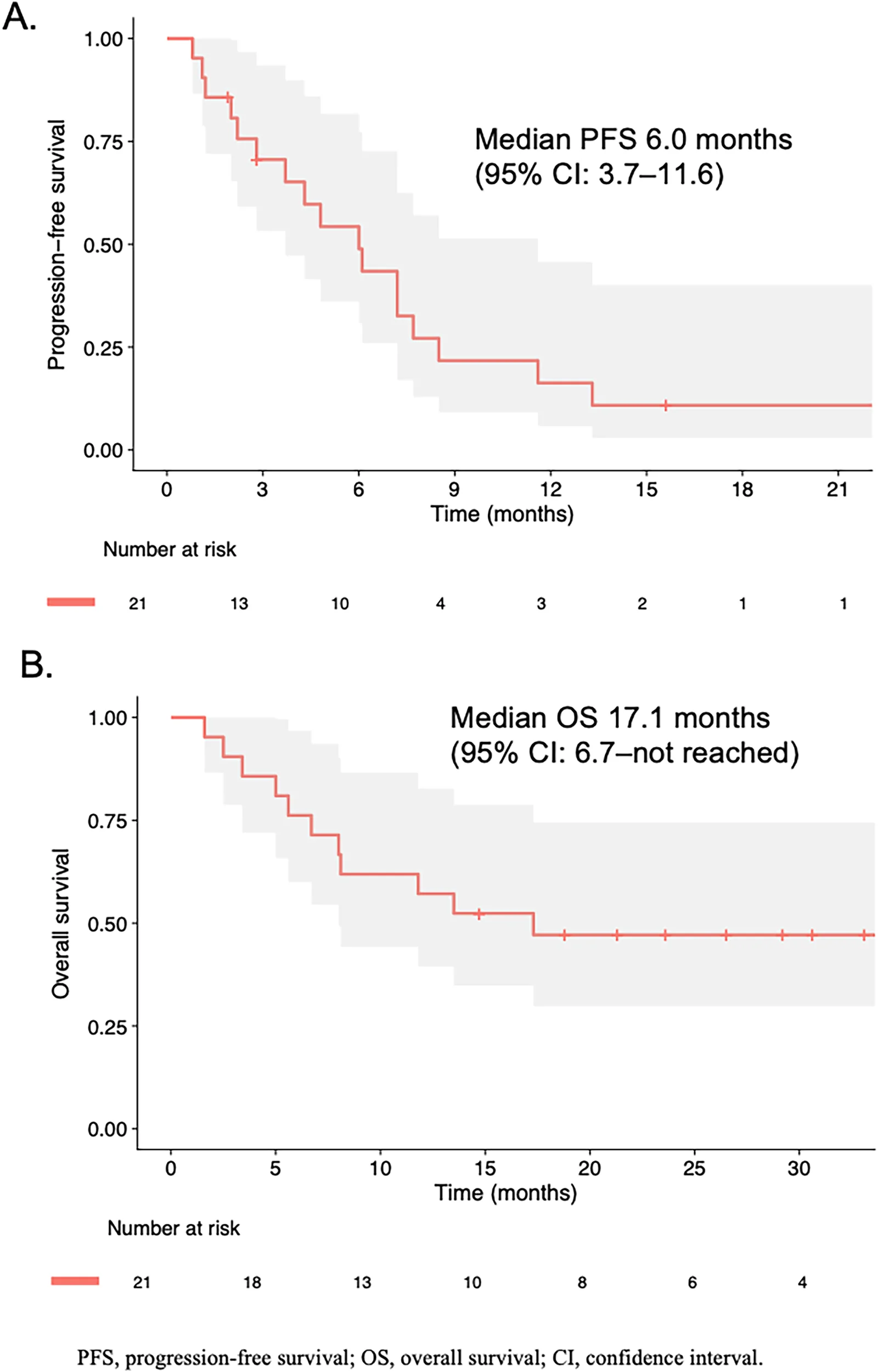
Kaplan–Meier curves for **(A)** progression-free survival and **(B)** overall survival in the efficacy population (n=21). PFS, progression-free survival; OS, overall survival; CI, confidence interval.

**Figure 4:**
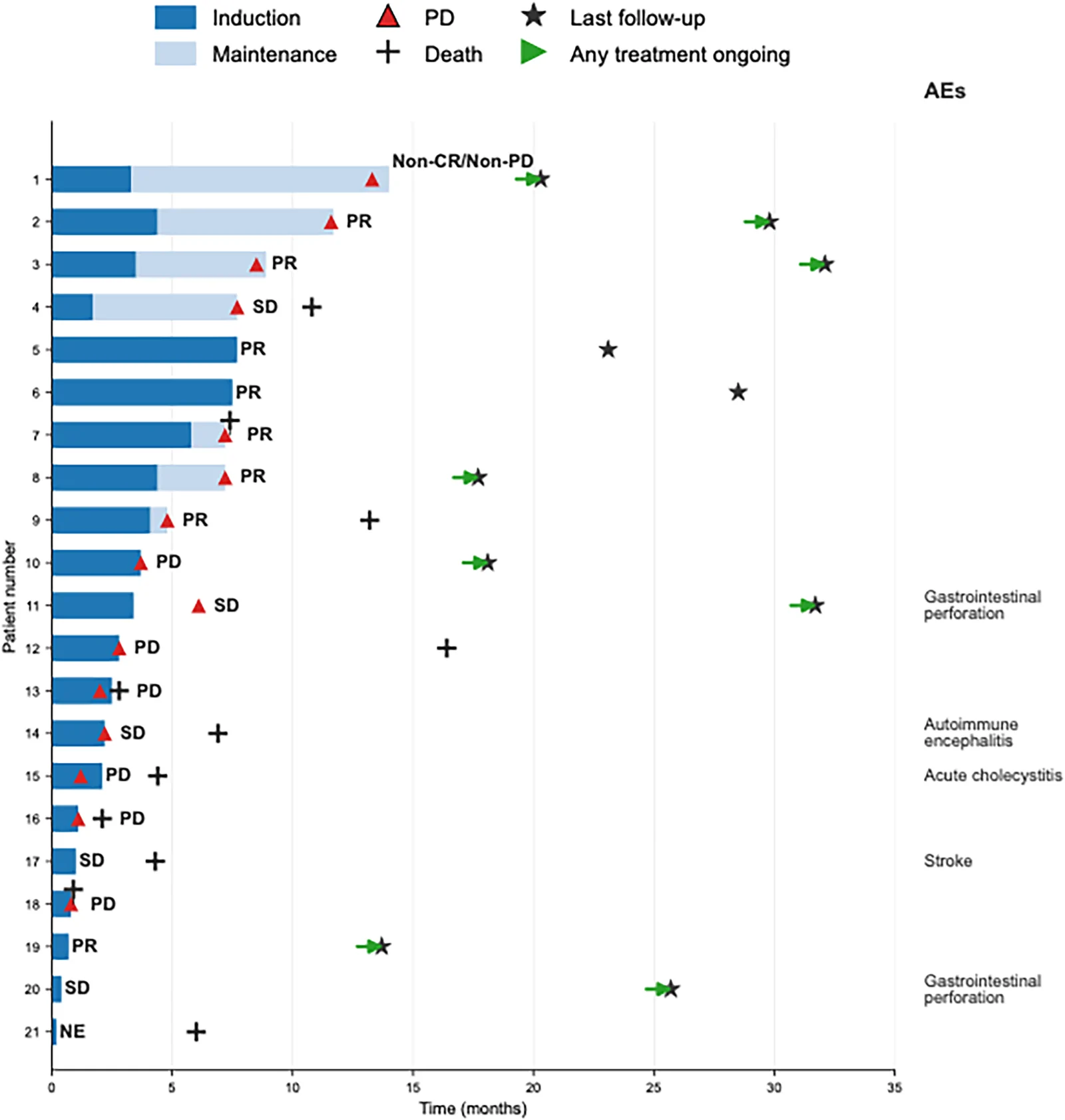
Swimmer plot of the efficacy population (n=21) showing individual treatment duration, tumor response, and key clinical events from the start of treatment. Each horizontal bar represents one patient, ordered from the longest to the shortest total treatment duration. Dark blue bars indicate ABCP induction therapy, and light blue bars indicate atezolizumab plus bevacizumab maintenance therapy. Best overall response is shown to the right of each bar. Red triangles indicate disease progression, black plus signs indicate death at the recorded time of death, black stars indicate last follow-up, and green arrows indicate that any systemic anticancer treatment was ongoing at the data cutoff. The right-hand column (AEs) lists adverse events that led to treatment discontinuation. AE, adverse event; PR, partial response; SD, stable disease; PD, progressive disease; NE, not evaluable.

### 3.3 Safety of ABCP in patients with *KRAS*-mutant NSCLC

In the safety population (n=21), grade ≥3 hematologic adverse events were observed in seven patients (33.3%), including neutropenia (n=4, 19.0%), leukopenia (n=3, 14.3%), febrile neutropenia (n=2, 9.5%), anemia, and thrombocytopenia (each n=1, 4.8%) (**Table 2**). Grade ≥3 non-hematologic adverse events were observed in 10 patients (47.6%), with hypertension, gastrointestinal perforation, and pneumonitis (each n=2, 9.5%), followed by anorexia, rash, autoimmune encephalitis, enthesitis, and stroke (each n=1, 4.8%). AEs leading to treatment discontinuation occurred in five patients (23.8%) and included gastrointestinal perforation in two patients (Grade 4 and Grade 3, respectively), autoimmune encephalitis in one patient (Grade 4), acute cholecystitis in one patient (Grade 2), and stroke in one patient (Grade 3) (**Figure 4**). All five AEs leading to treatment discontinuation were considered related to ABCP by the treating investigators. One patient discontinued ABCP after traumatic subarachnoid hemorrhage, which was unrelated to ABCP and was not classified as an adverse event, and subsequently died of disease progression at 6.0 months after treatment initiation. Three patients discontinued ABCP because of death due to disease progression at 2.8, 0.9, and 2.1 months after treatment initiation. None of the deaths was considered attributable to ABCP, and no Grade 5 treatment-related adverse events occurred.

**Table 2:** Grade ≥3 treatment-related adverse events in the safety population (n=21).

Item	Grade ≥3 adverse events, n (%)
Hematologic toxicity	
Any	7 (33.3%)
Neutropenia	4 (19.0%)
Leukopenia	3 (14.3%)
Febrile neutropenia	2 (9.5%)
Anemia	1 (4.8%)
Thrombocytopenia	1 (4.8%)
Nonhematologic toxicity	
Any	10 (47.6%)
Hypertension	2 (9.5%)
Gastrointestinal perforation	2 (9.5%)
Pneumonitis	2 (9.5%)
Anorexia	1 (4.8%)
Rash	1 (4.8%)
Autoimmune encephalitis	1 (4.8%)
Enthesitis	1 (4.8%)
Stroke	1 (4.8%)

### 3.4 Biomarker analysis of the baseline serum VEGF-A and VEGF-D

Baseline characteristics of patients who underwent pretreatment blood sampling (n=17) are shown in **Supplementary Table 2**. The median baseline serum levels were 0.35 ng/mL (range, 0.05–2.84) for VEGF-A and 1.28 ng/mL (range, 0.75–5.51) for VEGF-D. Patients were categorized into above-median and below-median groups using the sample median as the cutoff, with values equal to the median included in the above-median group. The baseline characteristics according to VEGF-A and VEGF-D status are shown in **Supplementary Table 3**. The proportion of current or former smokers was higher in the above-median groups than in the below-median groups for both VEGF-A (100% [9/9] vs 62.5% [5/8], p=0.08) and VEGF-D (100% [9/9] vs 62.5% [5/8], p=0.08). No statistically significant differences were detected in the other baseline characteristics, although these comparisons should be interpreted cautiously because of the small sample size.

Among patients with measurable target lesions and available pretreatment serum samples (n=15), 5 had PR, 4 had SD, and 6 had PD. Accordingly, the ORR and DCR were 33.3% (5/15; 95% CI, 15.2–58.3) and 60.0% (9/15; 95% CI, 35.7–80.2), respectively. The ORR and DCR did not significantly differ between the above-median and below-median VEGF-A groups (ORR, 22.2% vs 50.0%; p=0.33; DCR, 66.7% vs 50.0%; p=0.62) or between the above-median and below-median VEGF-D groups (ORR, 25.0% vs 42.9%; p=0.61; DCR, 75.0% vs 42.9%; p=0.31) (**Supplementary Figure 2A**).

In the biomarker population (n=17), 13 PFS events and 10 OS events were observed. No significant difference was observed in PFS between the above-median and below-median VEGF-A groups (4.3 vs 5.4 months; HR, 1.18; 95% CI, 0.39–3.57; log-rank p=0.76) (**Supplementary Figure 2B**), and no significant difference in OS was observed between these groups (10.8 vs 13.2 months; HR, 1.58; 95% CI, 0.44–5.61; log-rank p=0.48) (**Supplementary Figure 2C**). Similarly, PFS did not significantly differ between the above-median and below-median VEGF-D groups (4.8 vs 3.7 months; HR, 1.07; 95% CI, 0.36–3.23; log-rank p=0.90) (**Supplementary Figure 2D**), and OS also did not significantly differ between these groups (10.8 months vs not reached; HR, 2.14; 95% CI, 0.55–8.31; log-rank p=0.26) (**Supplementary Figure 2E**). Baseline serum VEGF-A and VEGF-D levels were strongly correlated (Spearman r=0.87; 95% CI, 0.66–0.95; p<0.001) (**Supplementary Figure 3**). In sensitivity analyses treating baseline serum VEGF-A and VEGF-D levels as continuous variables, neither biomarker was significantly associated with PFS or OS in the univariable Cox proportional hazards models. Adjustment for smoking status did not materially alter these findings. However, the estimates had wide confidence intervals and should be interpreted cautiously because of the limited sample size and number of outcome events (**Supplementary Figure 4**). No evidence of violation of the proportional-hazards assumption was found for the VEGF-A or VEGF-D Cox models.

## 4 Discussion

In this prospective multicenter study, we evaluated the efficacy and safety of ICI plus bevacizumab-based chemotherapy and explored the associations between pretreatment serum VEGF-A and VEGF-D levels and clinical outcomes in patients with *KRAS*-mutant advanced or recurrent non-squamous NSCLC. The cohort showed a median PFS of 6.0 months, which was the primary endpoint. Among patients with measurable disease, the ORR and DCR were 42.1% and 68.4%, respectively, and the median OS was 17.1 months. Pretreatment serum VEGF-A and VEGF-D levels were not significantly associated with clinical outcomes in this exploratory biomarker analysis. These findings should be interpreted alongside those of prior pivotal studies, recognizing the limitations of cross-trial comparisons. In several pivotal trials of metastatic non-squamous NSCLC without EGFR or ALK alterations, adding a PD-1/PD-L1 inhibitor to bevacizumab plus chemotherapy improved PFS and OS compared with bevacizumab plus chemotherapy alone (11, 12, 22, 23). In an exploratory subgroup analysis of *KRAS*-mutant tumors from IMpower150, the ABCP arm achieved a median PFS of 8.1 months and median OS of 19.8 months, with a numerically favorable trend compared with bevacizumab plus chemotherapy alone (5). However, direct comparison between the present cohort and the IMpower150 subgroup is not appropriate because of differences in study design, eligibility criteria, sample size, baseline characteristics, and treatment setting. In particular, the present cohort included a relatively high proportion of older patients and patients with an ECOG-PS of ≥1, but the extent to which these characteristics contributed to the observed outcomes cannot be determined in a single-arm study. Accordingly, the numerical differences from the IMpower150 subgroup should not be interpreted as evidence of inferior efficacy. Rather, our findings should be regarded as descriptive and exploratory prospective evidence regarding the clinical activity and safety of this regimen in a distinct, molecularly defined population.

The therapeutic rationale for ICI plus bevacizumab-based chemotherapy in *KRAS*-mutant NSCLC is supported by clinical evidence and the biological plausibility of the combination. Clinically, beyond the IMpower150 *KRAS*-mutant subgroup analysis, a recent network meta-analysis suggested favorable PFS for ICI plus bevacizumab-based chemotherapy among evaluated regimens in *KRAS*-mutant advanced NSCLC (24). However, indirect comparisons cannot establish regimen superiority without direct prospective comparative data. A recent retrospective real-world study found no significant additional benefit from adding bevacizumab to ICI plus chemotherapy in *KRAS*-mutant non-squamous NSCLC (25). Together, these findings indicate that the comparative role of bevacizumab-containing chemoimmunotherapy in *KRAS*-mutant NSCLC remains uncertain. Current first-line treatment options for *KRAS*-mutant NSCLC include several ICI-based approaches, and treatment selection should be individualized according to PD-L1 expression, clinical characteristics, disease burden, molecular background, expected toxicity, and patient preference (7, 8, 26). Accordingly, the present findings should be interpreted as evidence regarding one potential treatment approach rather than as support for a preferred regimen. *KRAS*-mutant tumors should not be considered a biologically uniform population. Differences in *KRAS* mutation subtype, co-occurring genomic alterations, and the tumor immune microenvironment may contribute to heterogeneous responses to ICI-based regimens (5–8, 27). Oncogenic *KRAS* signaling is associated with enhanced angiogenesis and an immunosuppressive tumor microenvironment, partly via upregulation of the VEGF-related pathway (28). VEGF blockade may augment antitumor immunity by improving vascular function and reducing immunosuppressive signaling (13, 29). These observations provide a biological rationale for further investigation of ICI plus bevacizumab-based chemotherapy in *KRAS*-mutant NSCLC.

In our exploratory analysis, we assessed whether pretreatment circulating VEGF-A and VEGF-D levels could identify patients who were more likely to benefit from ICI plus bevacizumab-based chemotherapy. In our cohort, neither VEGF-A nor VEGF-D was significantly associated with response, PFS, or OS. However, the small biomarker population and limited number of outcome events resulted in wide confidence intervals and limited statistical power. Sensitivity analyses treating VEGF-A and VEGF-D as continuous variables and adjustment for smoking status yielded similar findings. Therefore, these exploratory results should be interpreted cautiously and require validation in larger cohorts. Notably, these results do not necessarily conflict with those of prior biomarker studies. A previous clinical trial-based analysis suggested that lower baseline serum VEGF-A levels may identify patients who derive greater benefit from bevacizumab (14).

VEGF-D was included in an exploratory, non-prespecified biomarker analysis based on the hypothesis that it may contribute to alternative angiogenic signaling during VEGF-A-specific inhibition. Preclinical studies have suggested that suppression of VEGF-A signaling may be accompanied by increased VEGF-D expression or enhanced responsiveness to VEGF-C and VEGF-D, potentially allowing persistent angiogenic activity despite VEGF-A-specific blockade (17, 18). However, these observations have not established VEGF-D as a clinically validated biomarker of bevacizumab resistance. Accordingly, VEGF-D was evaluated as an exploratory marker rather than as a direct pharmacodynamic or predictive biomarker of bevacizumab.

Studies in the ramucirumab setting indicated a different role for VEGF-D (15). This discrepancy may partly reflect differences in VEGF pathway inhibition, as bevacizumab targets VEGF-A, whereas ramucirumab blocks VEGFR-2 signaling activated by multiple ligands, including VEGF-D (30). Therefore, findings concerning VEGF-D in the ramucirumab setting cannot be directly extrapolated to bevacizumab-based therapy. Differences among studies may also reflect variations in treatment context, assay strategy, patient population, and endpoint definition. In particular, a predictive biomarker for the addition of bevacizumab is conceptually distinct from a prognostic marker evaluated within a single-treatment cohort. These considerations suggest that the clinical relevance of circulating VEGF-related biomarkers may be treatment-context dependent.

In the univariable analyses, MPE and GPS were associated with shorter OS. These associations are more appropriately interpreted as prognostic rather than treatment-specific findings. Both variables may reflect underlying disease burden or systemic condition rather than selective sensitivity or resistance to ICI plus bevacizumab-based chemotherapy, consistent with previous reports linking GPS and MPE to poorer outcomes in advanced NSCLC (31, 32). Given the small sample size and limited multivariable adjustment, these findings should be interpreted cautiously. Future studies incorporating molecular co-alterations, inflammatory markers, metastatic patterns, and pleural disease are needed to determine whether these factors have predictive value beyond their established prognostic roles.

From a safety perspective, ICI plus bevacizumab-based chemotherapy was feasible in many patients, although clinically important toxicities were observed. AEs led to treatment discontinuation in 23.8% of patients, and gastrointestinal perforation occurred in 9.5%. Although this proportion was based on only two events and was therefore imprecise, gastrointestinal perforation is a recognized bevacizumab-related AE with an increased risk reported in a meta-analysis (33). Before initiating bevacizumab-containing therapy, clinicians should assess for clinical features previously associated with gastrointestinal perforation, including bowel obstruction or ileus, active intra-abdominal inflammation or gastrointestinal ulceration, bowel involvement by tumor, recent abdominal surgery, and relevant concomitant medications (34, 35). During treatment, new abdominal symptoms should prompt immediate clinical and radiologic evaluation. Detailed information on these gastrointestinal risk factors was not systematically collected in the present study, and the factors contributing to the two gastrointestinal perforation events therefore could not be determined. Careful patient selection and close monitoring remain important when bevacizumab-containing therapy is used.

This study has several strengths. Prospective multicenter data on *KRAS*-mutant non-squamous NSCLC treated with ICI plus bevacizumab-based chemotherapy remain limited, particularly in Japanese clinical practice settings. The prospective design, multicenter participation, and pretreatment serum sample collection provide prospective descriptive data that complement the existing literature. However, this study has several limitations. First, the small sample size, including only 21 patients in the efficacy and safety analyses and 17 patients in the biomarker analysis, limited statistical power and the precision of the effect estimates. The wide confidence intervals around several efficacy and biomarker estimates indicate substantial uncertainty. Second, the single-arm design without a control group precluded direct comparison of efficacy. Third, tumor response was assessed by investigators at each institution rather than through central independent review. Fourth, co-occurring genomic alterations, including *STK11*, *KEAP1*, and *TP53*, were not assessed. *KRAS*-mutant NSCLC comprises biologically and clinically heterogeneous subgroups defined by *KRAS* mutation subtype, co-occurring genomic alterations, and distinct tumor immune microenvironments (5–8). These molecular features may influence prognosis and responses to ICIs. Therefore, the lack of information on these co-occurring genomic alterations limited our ability to determine whether clinical outcomes differed among biologically distinct *KRAS*-mutant subgroups. Finally, the biomarker analysis was exploratory, was restricted to patients with available pretreatment serum samples, and used median-based dichotomization, which may have reduced statistical efficiency and obscured associations across the continuous biomarker range. Accordingly, the biomarker findings should be considered preliminary and require validation in larger, prospectively designed cohorts.

## 5 Conclusion

The results of this prospective multicenter cohort study showed an ORR of 42.1%, a median PFS of 6.0 months, and a median OS of 17.1 months with ICI plus bevacizumab-based chemotherapy in patients with advanced or recurrent *KRAS*-mutant non-squamous NSCLC. However, these findings should be interpreted as descriptive and exploratory because of the small sample size, single-arm design, and absence of a comparative cohort. Pretreatment serum VEGF-A and VEGF-D levels were not significantly associated with clinical outcomes in this exploratory analysis, although the limited sample size may have reduced the ability to detect clinically meaningful associations. Larger prospective studies with comparative designs and comprehensive molecular annotation are needed to validate potential biomarkers and to clarify the role of this regimen within the evolving first-line treatment landscape for *KRAS*-mutant NSCLC.

## Data Availability

The raw data supporting the conclusions of this article will be made available by the authors, without undue reservation.
