# Supplementary material for: Efficacy and safety of immune checkpoint inhibitor plus bevacizumab-based chemotherapy in advanced or recurrent KRAS-mutant non-squamous NSCLC: the SPIRAL-STONE study
Source: Front Immunol. 2026 Oct 12;17:1875501. doi: 10.3389/fimmu.2026.1875501 (PMC13696455; doi:10.3389/fimmu.2026.1875501)
Supplement: Supplementary file 1 [file DataSheet1.pdf]

Supplementary Material

Supplementary Figures

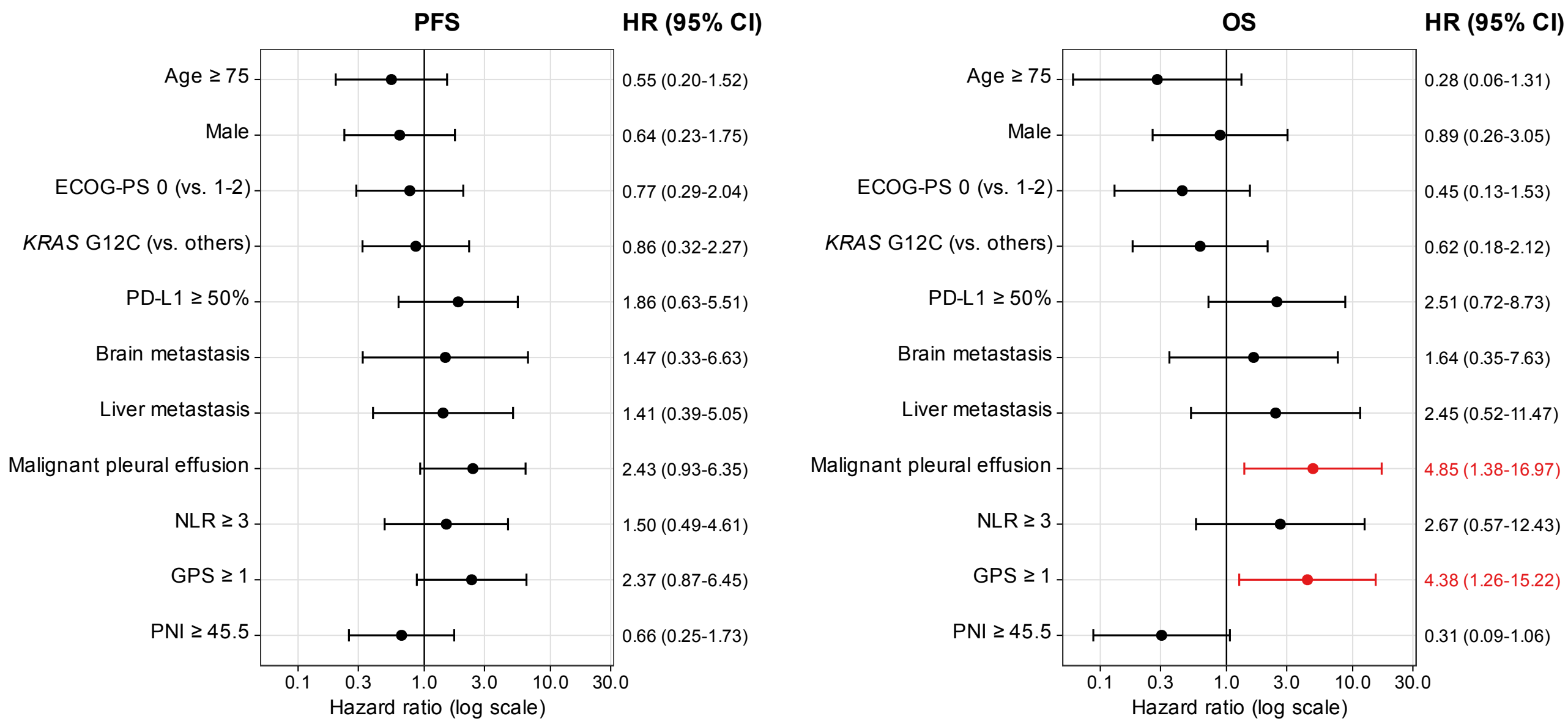

Supplemental Fig. 1. Univariable Cox proportional hazards analyses for PFS and OS. HRs with 95% CIs are shown on a logarithmic scale. Significant associations ($p<0.05$) are highlighted in red. PFS, progression-free survival; OS, overall survival; HR, hazard ratio; CI, confidence interval; ECOG-PS, Eastern Cooperative Oncology Group performance status; PD-L1, programmed death-ligand 1; NLR, neutrophil-to-lymphocyte ratio; GPS, Glasgow Prognostic Score; PNI, prognostic nutritional index; IHC, immunohistochemistry.

A.

	Responder, N	ORR (95% CI)	p-value	Disease control, N	DCR (95% CI)	p-value
Total (n = 15)	5	33.3% (15.2–58.3)		9	60.0% (35.7–80.2)	
VEGF-A						
Above-median (n = 9)	2	22.2% (6.3–54.7)	0.33	6	66.7% (35.4–87.9)	0.62
Below-median (n = 6)	3	50.0% (18.8–81.2)		3	50.0% (18.8–81.2)	
VEGF-D						
Above-median (n = 8)	2	25.0% (7.1–59.1)	0.61	6	75.0% (40.9–92.9)	0.31
Below-median (n = 7)	3	42.9% (15.8–75.0)		3	42.9% (15.8–75.0)	

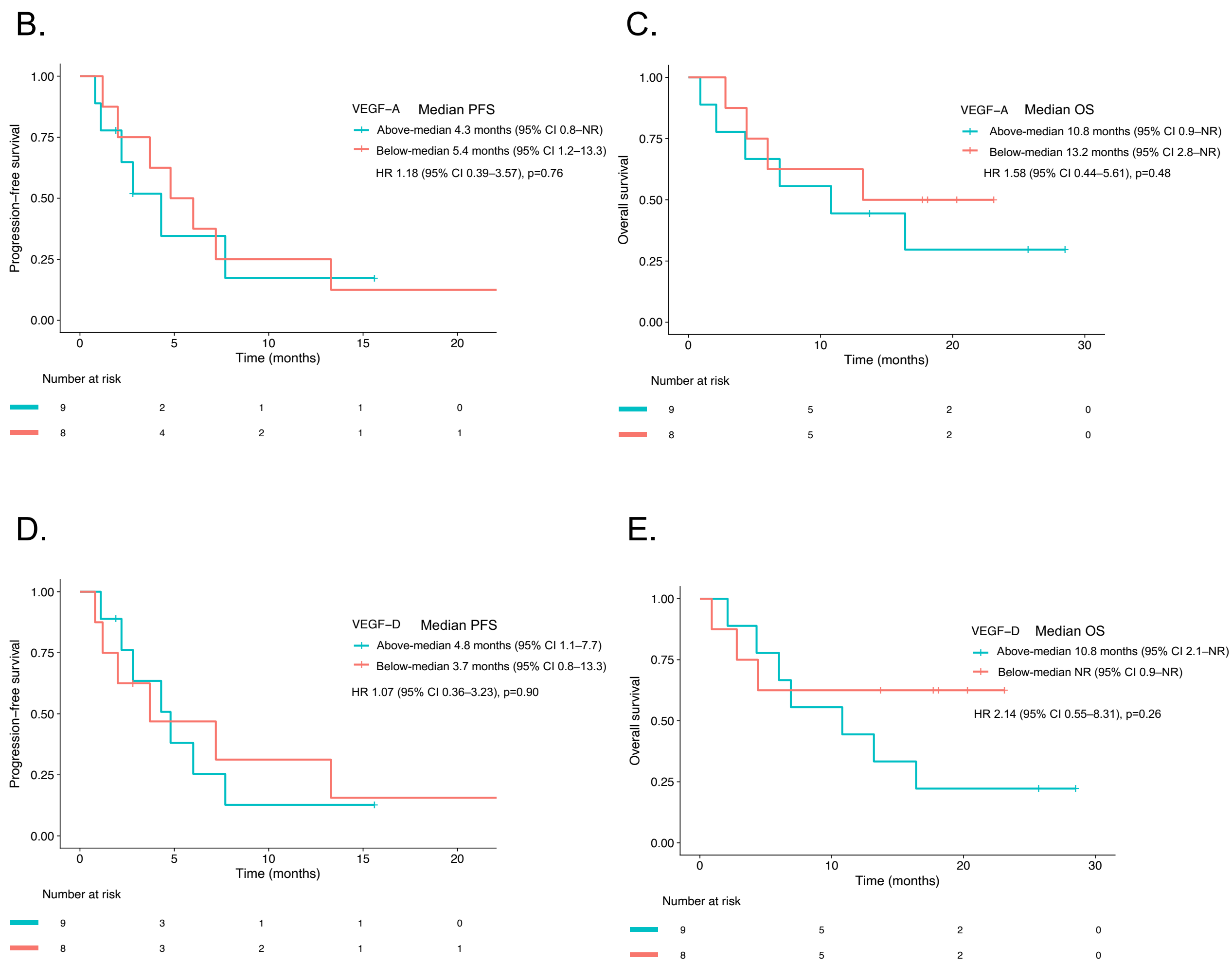

Supplemental Fig. 2. (A) ORR and DCR in the biomarker response-evaluable population with measurable target lesions (n=15), and Kaplan–Meier curves for PFS (B, D) and OS (C, E), stratified

according to above-median vs. below-median serum VEGF-A (B, C) or VEGF-D (D, E) levels in the biomarker population (n=17).

Above-median was defined as a value greater than or equal to the sample median (VEGF-A, 0.35 ng/mL; VEGF-D, 1.28 ng/mL), whereas below-median was defined as a value below the sample median.

ORR, objective response rate; DCR, disease control rate; PFS, progression-free survival; OS, overall survival; VEGF, vascular endothelial growth factor; HR, hazard ratio; CI, confidence interval; NR, not reached.

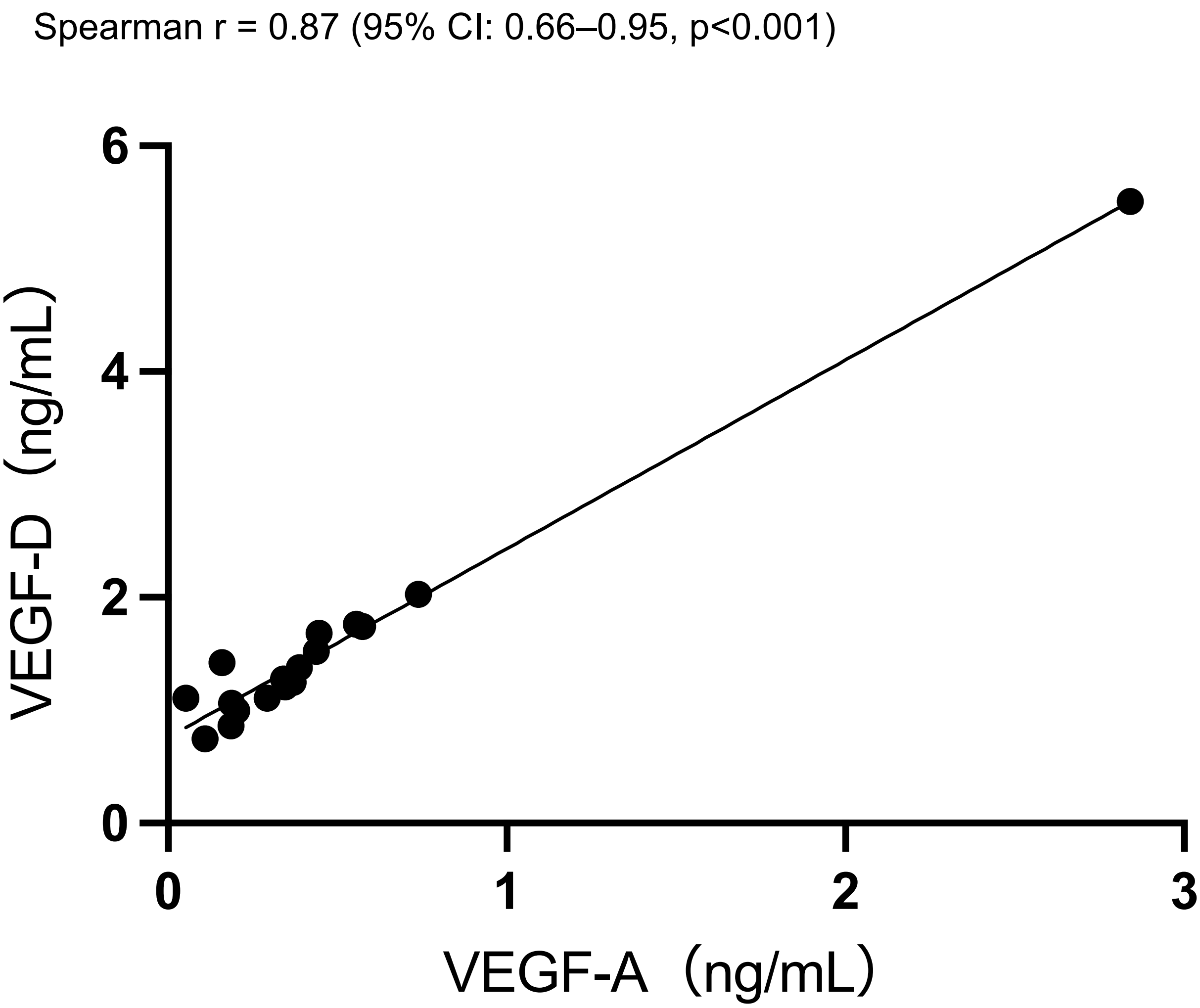

Supplemental Fig. 3. Correlation between VEGF-A (ng/mL) and VEGF-D (ng/mL) among patients who underwent blood sampling (n = 17). VEGF, vascular endothelial growth factor.

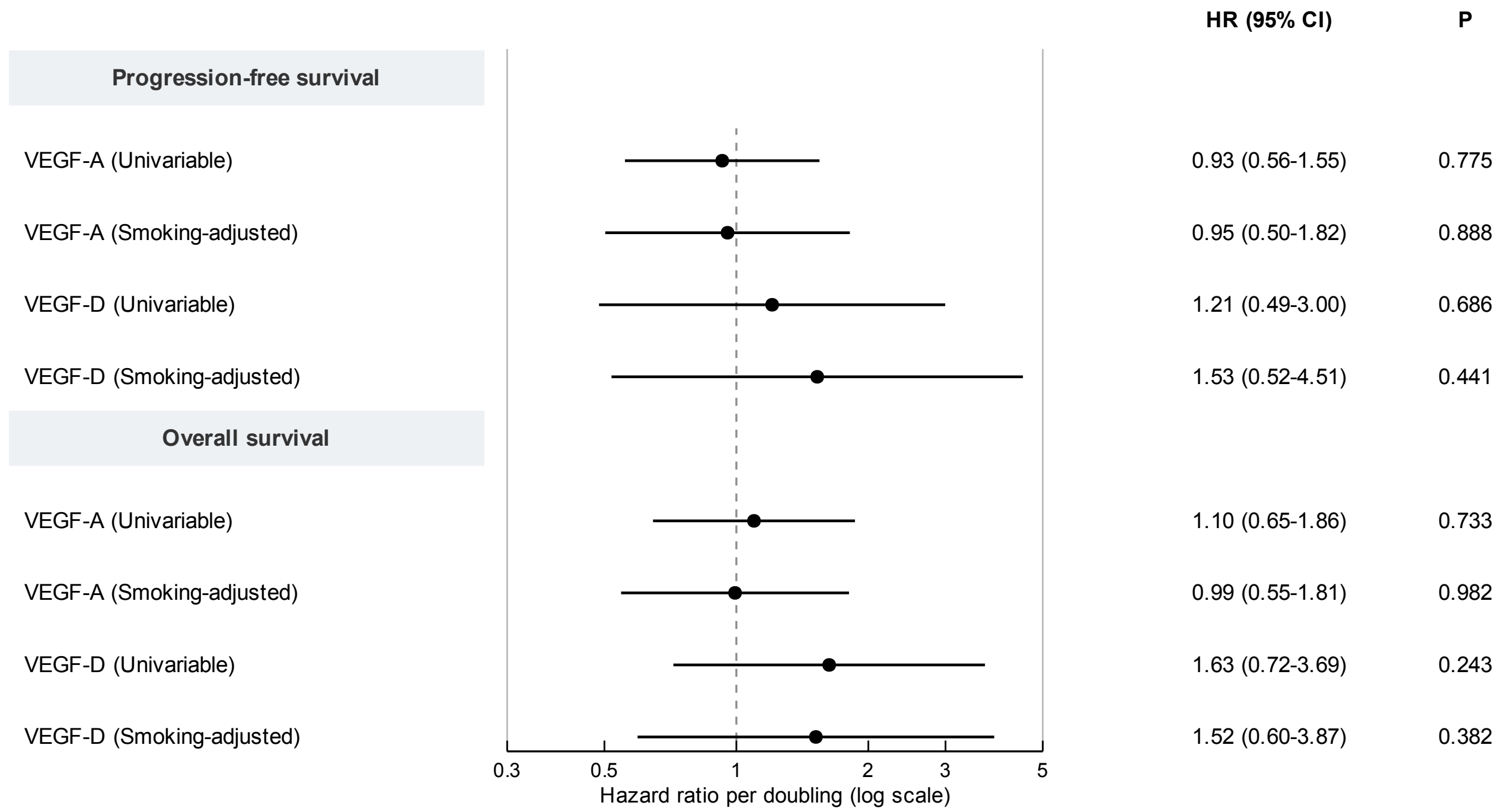

Supplemental Fig. 4. Associations of baseline serum VEGF-A and VEGF-D levels, analyzed as continuous variables, with PFS and OS. Hazard ratios are expressed per doubling of the biomarker level. Univariable estimates and estimates adjusted for smoking status are shown with 95% CIs. The vertical dashed line indicates a hazard ratio of 1.0.

VEGF, vascular endothelial growth factor; PFS, progression-free survival; OS, overall survival; HR, hazard ratio; CI, confidence interval.

1 Supplementary Tables

Supplemental Table 1. Subsequent systemic therapies after immune checkpoint inhibitor plus bevacizumab-based chemotherapy, summarized according to each patient and *KRAS* mutation subtype

Patient ID	*KRAS* mutation subtype	Subsequent systemic therapies after ICI plus bevacizumab based chemotherapy
1	G12C	Sotorasib
2	G12C	Sotorasib → Nab-paclitaxel → Pemetrexed + Bevacizumab
3	G12C	Sotorasib → Docetaxel + Ramucirumab → Atezolizumab + Carboplatin + Pemetrexed → Cisplatin + S-1
4	G12A	Pemetrexed
5	Others	None
6	Others	None
7	G12C	Sotorasib
8	Others	Docetaxel + Ramucirumab → Nivolumab + Ipilimumab
9	G12C	Sotorasib
10	Others	Nivolumab + Ipilimumab → Pemetrexed
11	G12D	Docetaxel → Carboplatin + Paclitaxel + Bevacizumab
12	G12A	Carboplatin + Pemetrexed + Bevacizumab
13	G12D	None
14	G12C	Sotorasib
15	G12C	None

16	Others	None
17	G12V	None
18	G12D	None
19	G12C	Sotorasib
20	G12C	Sotorasib → S-1
21	Others	None

KRAS, Kirsten rat sarcoma viral oncogene homolog; ICI, immune checkpoint inhibitor.

Others: variants reported collectively as "KRAS Others" by the AmoyDx assay, including G12A, G12V, G12R, and G13C; individual subtypes were not distinguishable using this assay, whereas variants detected by the Oncomine-based assay were reported individually.

Supplemental Table 2. Patient characteristics who underwent blood sampling (n = 17)

Characteristics	Patients (n = 17)
Age	
Median (range)	70 (48–81)
≥75	5 (29.4%)
Sex	
Male	11 (64.7%)
Female	6 (35.3%)
ECOG-PS	
0	8 (47.1%)
1	7 (41.2%)
2	2 (11.8%)
Tumor histology	
Adenocarcinoma	16 (94.1%)
Pleomorphic carcinoma	1 (5.9%)
Stage	
Unresectable stage III	2 (11.8%)
Stage IV	11 (64.7%)
Postoperative recurrence	4 (23.5%)

Metastasis site	
Bone	8 (47.1%)
Malignant pleural effusion	7 (41.2%)
Liver	3 (17.6%)
Brain	3 (17.6%)
KRAS mutation subtype	
G12C	6 (35.3%)
G12D	2 (11.8%)
G12A	2 (11.8%)
G12V	1 (5.9%)
Others	6 (35.3%)
PD-L1 TPS	
≥50%	5 (29.4%)
1–49%	6 (35.3%)
<1%	6 (35.3%)
Smoking status	
Current/Former	14 (82.4%)
Never	3 (17.6%)
Median serum levels (ng/mL, range)	
VEGF-A	0.35 (0.05–2.84)

VEGF-D	1.28 (0.75–5.51)

ECOG-PS, Eastern Cooperative Oncology Group performance status; *KRAS*, *Kirsten rat sarcoma viral oncogene homolog;* PD-L1, programmed death-ligand 1; TPS, tumor proportion score.

Others: variants reported collectively as “*KRAS* Others” by the AmoyDx assay, including G12A, G12V, G12R, and G13C; individual subtypes were not distinguishable using this assay.

Supplemental Table 3. Baseline patient characteristics and between-group comparisons based on serum VEGF-A or VEGF-D status among patients who underwent blood sampling.

	VEGF-A			VEGF-D		
	Above-median (n = 9)	Below-median (n = 8)	p-value	Above-median (n = 9)	Below-median (n = 8)	p-value
Age						
Median (range)	73 (48–77)	69 (57–81)	0.74	70 (48–77)	72 (57–81)	1.00
≥75	3 (33.3%)	2 (25.0%)	1.00	2 (22.2%)	3 (37.5%)	0.62
Sex						
Male	5 (55.6%)	6 (75.0%)	0.62	7 (77.8%)	4 (50.0%)	0.33
Female	4 (44.4%)	2 (25.0%)		2 (22.2%)	4 (50.0%)	
ECOG-PS						
0	3 (33.3%)	5 (62.5%)		4 (44.4%)	4 (50.0%)	
1	4 (44.4%)	3 (37.5%)	0.44	4 (44.4%)	3 (37.5%)	1.00
2	2 (22.2%)	-		1 (11.1%)	1 (12.5%)	
Tumor histology						
Adenocarcinoma	8 (88.9%)	8 (100%)	1.00	8 (88.9%)	8 (100%)	1.00
Pleomorphic carcinoma	1 (11.1%)	-		1 (11.1%)	-	
Stage						

Unresectable stage III or IV	7 (77.8%)	6 (75.0%)	1.00	6 (66.7%)	7 (87.5%)	0.58
Postoperative recurrence	2 (22.2%)	2 (25.0%)		3 (33.3%)	1 (12.5%)	
Metastasis site						
Bone	4 (44.4%)	4 (50.0%)	1.00	3 (33.3%)	5 (62.5%)	0.35
Malignant pleural effusion	4 (44.4%)	3 (37.5%)	1.00	4 (44.4%)	3 (37.5%)	1.00
Liver	1 (11.1%)	2 (25.0%)	0.58	-	3 (37.5%)	0.08
Brain	3 (33.3%)	-	0.21	2 (22.2%)	1 (12.5%)	1.00
KRAS mutation subtype						
G12C	3 (33.3%)	3 (37.5%)	1.00	3 (33.3%)	3 (37.5%)	1.00
Non-G12C	6 (66.7%)	5 (62.5%)		6 (66.7%)	5 (62.5%)	
PD-L1 TPS						
≥50%	3 (33.3%)	2 (25.0%)		2 (22.2%)	3 (37.5%)	
1–49%	3 (33.3%)	3 (37.5%)	1.00	4 (44.4%)	2 (25.0%)	0.84
<1%	3 (33.3%)	3 (37.5%)		3 (33.3%)	3 (37.5%)	
Smoking status						
Current/Former	9 (100%)	5 (62.5%)	0.08	9 (100%)	5 (62.5%)	0.08
Never	-	3 (37.5%)		-	3 (37.5%)	

Above-median was defined as a value greater than or equal to the sample median (VEGF-A, 0.35 ng/mL; VEGF-D, 1.28 ng/mL), whereas below-median was defined as a value below the sample median.

VEGF, vascular endothelial growth factor; ECOG-PS, Eastern Cooperative Oncology Group performance status; *KRAS*, *Kirsten rat sarcoma viral oncogene homolog;* PD-L1, programmed death-ligand 1; TPS, tumor proportion score.
